# Informed consent in the psychosis prodrome: ethical, procedural and cultural considerations

**DOI:** 10.1186/1747-5341-9-19

**Published:** 2014-11-18

**Authors:** Sarah E Morris, Robert K Heinssen

**Affiliations:** Division of Adult Translational Research, National Institute of Mental Health, 6001 Executive Blvd, North Bethesda, MD 20892 USA; Division of Services and Intervention Research, National Institute of Mental Health, 6001 Executive Blvd, North Bethesda, MD 20892 USA

**Keywords:** Ethics, Informed consent, Prodrome, Psychosis, Schizophrenia, International, Culture

## Abstract

Research focused on the prodromal period prior to the onset of psychosis is essential for the further development of strategies for early detection, early intervention, and disease pre-emption. Such efforts necessarily require the enrollment of individuals who are at risk of psychosis but have not yet developed a psychotic illness into research and treatment protocols. This work is becoming increasingly internationalized, which warrants special consideration of cultural differences in conceptualization of mental illness and international differences in health care practices and rights regarding research participation. The process of identifying and requesting informed consent from individuals at elevated risk for psychosis requires thoughtful communication about illness risk and often involves the participation of family members. Empirical studies of risk reasoning and decisional capacity in young people and individuals with psychosis suggest that most individuals who are at-risk for psychosis can adequately provide informed consent; however ongoing improvements to tools and procedures are important to ensure that this work proceeds with maximal consideration of relevant ethical issues. This review provides a discussion of these issues in the context of international research efforts.

## Introduction

Evidence that increased duration of untreated psychosis is related to worse outcomes, including delay in medication response [1], more severe symptoms [2], and earlier relapse [3], has invigorated clinical research efforts to identify individuals who are likely to develop psychosis prior to the onset of full-blown illness. Accumulated data now clearly indicates that psychosis onset is typically predated by a prodromal phase which is of variable length and starts in early adolescence or late childhood. Research approaches focused on individuals who are variously described as ultra-high risk (UHR), clinical high risk (CHR), or having at-risk mental state (ARMS) have allowed researchers to address the psychopathological and neurobiological underpinnings of the putative prodromal state for psychosis and to test new interventions aimed at preventing or delaying the development of psychosis.

Further work is needed to improve predictive accuracy, understand underlying neurobiological mechanisms, and develop rational strategies for interventions aimed at pre-empting illness. This ambitious research agenda will entail enrolling large numbers of individuals who are at risk of psychosis into research protocols. Much of the prior work on psychosis risk has been conducted in Western countries but a crucial aspect of future work will be to examine similarities and differences in the psychosis trajectory among different countries and cultures. Expanding this research agenda in non-Western countries confers at least two scientific advances: it allows the scientific community to examine the ways in which the patterns of risk and outcomes vary among countries and allows larger, more definitive and representative studies. International expansion of psychosis risk research requires careful consideration of the ethical and cultural issues involved in obtaining informed consent from young adults judged to be at heightened risk for psychotic illness. Differences in economic and healthcare opportunities, scientific worldview, and institutional practices among countries and cultures require careful consideration of ethical research practices and use of consent documents and procedures that take into the consideration the economic, linguistic and social characteristics of the individual and the local environment [4]. In this article, we review research relevant to these issues and make suggestions regarding the informed consent process.

## Review

### Identifying at-risk individuals

A number of clinical criteria have been suggested and tested to identify subjects with an increased risk of developing psychosis within a relatively short period of time [5, 6]. Converging evidence shows that individuals at high clinical risk for psychosis who present with sub-threshold and attenuated psychotic symptoms and signs have an enhanced liability for developing a psychotic disorder over time. For some of these individuals, these sub-syndromal symptoms will prove to be early manifestations of psychotic illness. For others, the symptoms will persist without exacerbation and may not become the focus of clinical attention or may resolve entirely over time. A recent meta-analysis of more than 2500 high risk subjects quantified such vulnerability, showing a transition risk that increased from 18% after six months to 36% after three years following first presentation [7]. Notably, most of the subjects who later develop a psychotic illness will transition to an ICD/DSM schizophrenia spectrum psychosis (73%) rather than to an affective psychotic illness (11%, Risk Ratio = 5.4) [8]. The high risk state is usually associated with distressing symptoms, impaired quality of life and subtle, albeit significant, deficits in cognitive functioning [9]. At a neurobiological level, there are alterations in brain structure [10, 11], function [12], connectivity [13] and neurochemistry [14–17]. Some of these brain abnormalities are associated with the high risk state, while others are dynamic predictors of the longitudinal development of psychosis [18]. New treatments to reduce the disability associated with sub-threshold symptoms or to prevent the frank onset of illness are being investigated [19, 20].

Differences in health care systems and patterns of referral for specialized care affect the ways in which high risk individuals can be identified for research purposes and the implementation of intervention research. Base rates of self-reported psychosis prodrome symptoms appear to differ among countries [21, 22], thus impacting estimates of prevalence and conversion rates. Translation and validation of the measures used to detect the psychosis risk state facilitates data sharing and integration across countries and cultures. For example, the Structured Interview for Prodromal Syndromes (SIPS) has been translated into Korean and validated with a sample of 40 subjects, 12.5% of whom developed psychosis during a one-year follow-up period [23]. Reliability of the SIPS has also been tested in Japan [24]. Another measure, the PRIME screening tool, has been studied in a variety of countries and languages, including Kenya [25], Mexico [22], and Japan [26]. Further work is needed to determine the extent to which differences in rates of psychosis risk symptoms among countries reflect actual variation in prevalence or are due to differences in screening procedures, willingness to report symptoms, or other factors.

### Communicating the nature of the prodrome

At the time of recruitment into a prodromal psychosis study, many high risk patients are already experiencing depression, anxiety, changes in thinking, problems in basic cognition, and social impairment [27–31]; some may already be receiving psychosocial or pharmacological [32] treatment. For these individuals, questions about diagnosis, likely outcomes, and treatment options are common. For others who may not previously have thought of themselves as being at risk of mental illness, the invitation to participate in a psychosis risk study is likely to stimulate similar questions. Heinssen and colleagues [32], summarizing the results of an NIMH-sponsored workshop on informed consent in early psychosis research, recommended three specific “informed consent bullets” that should be communicated to potential participants regarding the nature of psychosis risk:

1. Individuals who are at increased risk of psychosis compared to the general population can be identified with acceptable reliability by using structured instruments administered by trained personnel. Among people who meet at-risk criteria, however, we are currently unable to predict precisely who will go on to develop a psychotic disorder and who will not.
2. Transition to psychosis is not inevitable or predetermined for individuals who are identified as being as risk. Research suggests that about 36% of individuals who meet clinical risk criteria will develop psychosis within three years [33]. Psychosis risk for any individual will vary depending on factors such as family history, symptom severity, and change in functioning but it is important to communicate that serious mental illness is not foreordained. However, independent from the risk of developing a psychotic disorder, vulnerable subjects often experience co-occurring problems and disability which can be more distressing than prodromal symptoms themselves. Thus, it is important to inform prospective research participants about outcomes other than psychosis in the process of requesting informed consent.
3. At present, we do not have enough research to definitively recommend specific treatments for persons who are at risk for psychosis. Findings from pharmacotherapy studies do not support broad use of antipsychotic medications as a first-line treatment approach with at-risk individuals [20]. However, cognitive behavioral therapy, family work, substance abuse prevention, and cognitive remediation are among promising psychological treatment approaches that appear effective during the earliest stages of the prodrome [34]. All of these options, as well as other reasonable medication and psychosocial interventions for treating subjects’ presenting problems, should be disclosed to potential subjects as potential alternative treatment options [35].

In summary, the decision to provide consent should be informed by a full explanation of what is currently known about the psychosis prodrome, specifically, that many individuals who are at clinical risk do not develop psychosis, and that among those who don’t, some have complete resolution of symptoms while others may develop disorders other than psychosis [36–38]. It is equally important to convey what is not known, most importantly that it is not possible to predict whether any given individual will eventually develop psychosis. Given the range of difficulties observed among at-risk individuals, however, potential research participants should also understand that a potential benefit of research participation includes the possibility of receiving early and effective intervention should a disorder develop, and the possibility of reduced distress from the prodromal or at-risk symptoms per se [39].

### Competence, capacity and coercion

Studies of the psychosis prodrome require large samples of high risk individuals who are willing to be followed over considerable periods of time. Both up-front refusal and downstream attrition threaten the quality and quantity of research in this area, but it is paramount that the informed consent process be conducted in a non-coercive manner. Many of the issues related to consent processes apply broadly to all areas of medical research but others are more specifically relevant to psychosis risk research due to cultural differences in ideas about mental illness, stigma, the involvement of predominantly adolescent individuals and the role of the family in consent and research processes. A full review of these shared and specific considerations is beyond the scope of this review but a selective review is provided here.

Cultural differences in doctor-patient relationship and communication [40], attitudes toward personal autonomy and individualism, and institutional trust, and language and social class differences [41, 42] should be taken into consideration when formulating procedures related to obtaining informed consent so that potential participants may feel fully free to ask questions about the study and to decline to participate. In Japan, as an example, there is an effort to increase the use of informed consent for medical procedures and reduce paternalism in the doctor-patient relationship while preserving the autonomy of the physician [43]. It isn’t always possible to reduce real or perceived power or status differentials between study staff and potential participants and methods for addressing these factors which may affect the consent process will vary depending on the details of the research project and the setting. Care should, of course, be taken to ensure that informed consent documents can be understood by all potential participants. To maximize the exercise of personal choice in the consent process in cultures in which deference to authority is high, for example, research staff may want to avoid having a physician who is in a leadership role in the study approach potential participants for consent.

Specific to studies of mental illness, culture-based differences in understandings of psychiatric symptoms [44] might impact potential participants’ reasoning about study participation. For example, spiritual attributions about the causes of mental disorders are prevalent in many cultures [45, 46] or individuals may have a combination of biomedical and indigenous beliefs about the causes and treatment of mental disorders [47]. International differences in laws regulating the treatment of persons with mental illness affect procedures for enrolling individuals in research protocols. For example, in Japan and Korea, obtaining informed consent for treatment of psychiatric patients is a relatively new practice and, as a result, novel measures for assessing competency to consent to treatment have been developed [48, 49], which could be also useful for research purposes.

Informed consent for research participation is predicated on the assumption that the prospective participant is competent to give consent as evidenced by the ability to (1) understand relevant study information, including the reasons why they are being asked to participate, the procedures that they will be asked to undergo, and how to discontinue their participation); (2) appreciate the nature and consequences of accepting or declining the invitation to participate; (3) manipulate information rationally while making decisions; and (4) communicate a choice [50, 51]. Given the complexity of most informed consent documents and many research studies, it is not surprising that misunderstanding of consent documents and procedures such as randomization and blinded assignment to experimental groups and misconceptions about the purposes of research (i.e., the “therapeutic misconception”) are widespread [52]. Many instruments for assessing understanding of study information and decisional capacity have been developed [53] and they vary in their content, format, standardization, administration and psychometrics [54]. Different standards and cutoff scores have been used to determine decisional capacity in studies of individuals with psychosis, and although it is appropriate for stringency to differ depending upon the nature of the study-associated risks, it is important for investigators to consider *a priori* the impact of implementing different criteria on the screening and capacity determination outcomes [55]. It is also important to keep in mind that if a patient has capacity to provide consent for one type of study or intervention, it does not necessarily mean they have capacity to consent for other procedures [56] and that different measures of consent capacity will be appropriate for different settings and studies, depending on factors such as the degree of risk involved in the study procedures and the participants’ illness severity [54].

Competence and capacity for providing informed consent among individuals at risk for psychosis has rarely been the focus of empirical research, however, the results of an increasing number of studies of these processes in people with psychosis [57] may be informative for shaping policies and procedures for prodrome studies. Evidence that most schizophrenia patients are competent to provide informed consent [58–61] and anecdotal reports from large studies of at-risk individuals [62] suggest that most individuals who are at clinical or genetic risk for psychosis are competent to provide consent. There is, however, substantial variability in decisional capacity among schizophrenia patients and non-patients [61], so it must be assumed that at least some candidates for psychosis risk studies may not meet these standards for competence. Poor performance on a measure of decisional capacity has been associated with cognitive impairment and, to a lesser degree, with positive and negative symptoms of psychosis [55, 60].

Various types of educational efforts to enhance decisional capacity in individuals with schizophrenia have demonstrated efficacy, with some interventions successfully eliminating the disparity between patients and non-patients in understanding the elements of informed consent [63]. Thus, when considering whether a potential participant is capable of consenting to participate, the staff administering informed consent may want to consider whether diminished cognition or sub-syndromal symptoms are affecting decisional capacity and whether an enhanced consenting process with additional educational content might be appropriate.

When research is conducted in a clinical setting or with individuals who are already engaged with clinical systems, it is important for investigators to clearly describe which procedures are being done as part of the research protocol rather than clinical care and for participants to understand whether and how their decision to participate in research will affect their clinical care. It can be a challenge when clinical staff are assisting with subject recruitment, assessment or treatments, but participants should clearly understand the roles of the clinical and research staff members and effort should be made to avoid giving participants the impression that clinicians are pressuring them to provide consent for research participation.

Increasingly, research on psychosis risk has focused on the possible impact of immigration and status as a social minority [64]. Such studies require participation of immigrants and/or family members of immigrants, who might have cultural norms that are different than the dominant norms in their country of residence and might be less aware of their rights (both to participate and to decline participation) and more vulnerable to coercion [41]. When outreach and informed consent procedures are developed for use in these populations, particular care should be taken to ensure that potential participants understand the boundaries between study personnel and government officials. Studies may consider coordinating outreach efforts through cultural groups and organization in the local community in order to overcome cultural barriers to research participation [65].

With its massive population centers well-suited for the recruitment of large samples of at-risk individuals, several new research projects on the psychosis prodrome have focused their efforts in China. Pointing to the feasibility of such studies, methods used for identification and enrollment of at-risk individuals have been successfully adapted and implemented in a Chinese setting to recruit a sample with characteristics, including conversion rates, that are similar to those in large psychosis risk studies in other countries [66]. The new national Mental Health Law of China emphasizes de-stigmatization, prevention, and patient rights [67]. The law’s provision that inpatient treatment of mental disorders “shall generally be voluntary” reflects a change from the previously widespread assumption that psychiatric patients are unable to make decisions regarding inpatient treatment and the nearly ubiquitous practice that guardians made decisions regarding treatment [68]. In a study of consent competence among inpatients with schizophrenia in Hunan province, China, 28% of patients with schizophrenia were classified as competent to provide consent on the basis of their score on the Semi-structured Inventory for Competence Assessment [69], compared to 40% in a similar study conducted in London [70]. Despite a substantial minority of patients with capacity to consent, all of the consent documents for inpatient treatment for study participants had been signed by guardians. Among those, 18% were signed by both the guardian and the patient; however, patient co-signature was not related to patient score on the capacity assessment. Although this analysis focused specifically on consent for treatment, similar challenges in defining and determining capacity to consent are present for research endeavors.

### Risk reasoning in adolescence and psychosis

Studies of risk reasoning and decision-making in adolescents and individuals with psychosis can inform the process of soliciting informed consent with individuals who are at-risk of developing schizophrenia. Decision-making in adolescents may be impacted by greater responsiveness to peer pressure, altered risk perception, and increased focus on short-term risks and benefits [71]; these tendencies can affect adolescents’ thinking about their risk of developing illness as well as their risk of experiencing discomfort or adverse outcomes related to participation in a research study or clinical intervention. Variability in the rates of conversion reported in the research literature adds to the challenge in weighing the potential risks and benefits of research participation. A review of the empirical research concerning children’s competence to provide consent or assent for psychological and medical treatment and research [72] indicates that some aspects of treatment or research are understood by children more easily than other aspects and that understanding increases with age and when understanding is queried more frequently. The Institute of Medicine [73] provides guidance for ensuring appropriate levels of decisional support when involving young people in research.

There is evidence that individuals with schizophrenia have a propensity to value smaller rewards that are received quickly more than larger rewards delivered further into the future [74] and that they experience a foreshortened future time horizon [75]. Decision-making differences in individuals with early-onset schizophrenia, characterized by increased sensitivity to reward and decreased sensitivity to future outcomes, mirror these findings [76]. Cannabis abuse, a risk factor for schizophrenia [77, 78], has been associated with impaired decision making among first-episode patients with schizophrenia-spectrum psychosis [79].

Although it is not known whether these characteristics of risk reasoning are also present during the prodromal period, it is possible that individuals who are asked to participate in studies of the psychosis prodrome may tend to downplay the long-term risks and benefits of participation. Monetary (or other) incentives may be more highly salient for potential participants who have already experienced a decline in functioning than for less impaired individuals with more opportunities for financial income. Care should be taken to set compensation at a level that is reasonable and fair but not excessive to the point that it could be considered to be coercive. This is an especially important issue for research that is being conducted in low-income countries where some participants may be vulnerable to coercive payments due to a shortage of other opportunities for paid work. In contrast, some culturally Asian research participants might feel uncomfortable accepting payment for research participation because they are not accustomed to being paid by clinical personnel and payments may arouse suspicion about the purpose of the project [80].

### Involving families in informed consent

Research involving individuals at risk for psychosis may include children as young as 12 years old as well as many adolescents younger than 18. In these circumstances, adult family members often play a critical role in subject recruitment and consent activities.

Most studies rely on individuals’ self-report of symptoms and experiences to determine eligibility for research participation and for tracking changes in clinical status and functioning. There is, however, evidence that patient reports of functioning might be less accurate than those of high-contact clinicians, friends or family members [81]. Using a new caregiver-report version of the 12-item Prime Screen-Revised, investigators found that parent–child agreement about adolescents’ psychosis risk symptoms was poor, and that incorporating parents’ ratings improved the specificity of at-risk classification [82], indicating the potential utility of gathering input from collateral informants.

Policies regarding consent procedures differ among countries and institutions [73] but, generally, before an adolescent can participate in research, informed consent from a parent and assent from the at-risk adolescent is required. At the time of recruitment, some family members may be uninformed about the nature of mental disorders and may have the incorrect impression that they are to blame for their family member’s risk status. Family members typically react with distress upon being informed that their child is at increased risk of developing psychosis [62, 83] and sometimes appear reluctant to engage with research or clinical staff members. As recommended above for at-risk individuals, family members should be provided with accurate and up-to-date information about mental illness risk and the details of the project for which consent is being solicited. Referrals to sources of information about mental health and clinical support for family members should be made available as appropriate.

Parental attitudes can impact medical decisions made by adolescents and young adults [84] and family members often play an important role in research endeavors by helping to make sure that participants attend appointments and adhere to study protocols. Furthermore, parental attitudes, behavior, and coping strategies are associated with functional outcomes of at-risk youth [85] and degree of caregiver distress [86]. Despite concerns about the potentially stigmatizing effects of early identification, the majority of caregivers for psychiatrically ill family members report that they would have made use of clinical services for early detection if they had been given the opportunity, and believe that earlier intervention benefited both their ill family member and themselves [87]. A small, U.S.-based study suggests that levels of stigma are generally low among family members of prodromal individuals but relatively higher among ethnic minority families [88]. Research focused on the consent process is limited, but Li and Seidman [89] provide a several suggestions regarding family-related issues that may be involved when working clinically with Asian-American youth at high risk for psychosis which could apply to the research consent process. To address concerns regarding stigma, the informed consent process should include discussion of when clinical information about the adolescent will be shared or kept confidential and the circumstances under which family members will be notified about clinically significant events like suicidal ideation or intent. Family members should feel supported by the research team regardless of the decisions they make regarding research participation.

### Sustained consent, clinical contingencies and termination

Because research projects involving at-risk participants may have multi-year durations and participants’ willingness and capacity to provide consent may change during their involvement, it is important to ensure that informed consent is sustained throughout the period of participation. In an analysis of longitudinal consent-related abilities in research participants with schizophrenia [90], most participants’ capacity to understand study procedures either stayed steady or improved over time, however, understanding declined in approximately one quarter of the participants and 4% of participants failed to meet the initial capacity threshold at one or more follow-up assessment time point. These findings suggest that it isn’t necessary to re-evaluate consent capacity for all participants in longitudinal studies. However, predictors of decline in consent-related abilities like poor cognitive performance at baseline or worsening positive and negative symptoms over time, suggest the need to re-assess both capacity and consent whenever changes in clinical status are observed. Sustained consent procedures should include specification of the means by which participants may inform study staff if they wish to discontinue their participation.

One potential benefit of enrolling in a research protocol for at-risk individuals is that ongoing monitoring of clinical symptoms is often provided, allowing rapid detection of symptom escalation. Depending on the nature of the research activity, staff may need to consider whether it is appropriate to develop a plan to provide clinical referrals for persons who are screened for recruitment but, upon further evaluation, do not meet the study’s eligibility criteria. This plan should take into consideration the practicalities of the local health care system. A substantial proportion of individuals enrolled in studies of psychosis risk have previously been diagnosed with a mood or anxiety disorder [66, 91] and so ongoing treatment needs should be taken into consideration when planning for research participation. Information about contingencies for handling conversion to full-blown illness or other psychiatric crises should be included in the informed consent document and discussion and should take into consideration the points of entry into care systems that are available to the individual. Some studies allow psychotic individuals to continue in the protocol while others discontinue participation upon development of full-blown illness, with referral of psychotic individuals to other care providers. Medication trials with a blinded condition may discontinue the blind when psychotic symptoms are observed and immediately initiate individualized treatment. Similarly, it is important for protocols to include a plan for appropriate continuity of care for participants at the conclusion of their study participation. The issue of duration and discontinuation of treatment in longitudinal studies of psychosis risk is a challenging one because there is, at present, no method for determining whether an individual who does not develop psychosis during the course of a study will never go on to develop psychosis (*i.e*., a false positive) and can safely withdraw from treatment or are responsive to the intervention and likely to develop illness if intervention is discontinued [92]. Care should be taken to avoid inadvertently conveying during these discussions that the development of psychosis is inevitable or even more likely than not. Non-psychosis outcomes that require clinical attention are, however, common among participants in psychosis risk studies [36]. Depending on participants’ clinical status, post-study follow-up might involve referral for ongoing treatment or education regarding warning signs and self-monitoring for individuals who are asymptomatic or experiencing subthreshold symptoms.

Beyond the issues of consent and clinical contingencies for individual participants, researchers who are conducting their work in low-income countries in which information about mental illness and resources for treatment may be limited may want to consider ways in which they can incorporate the benefits of research into the local community via educational outreach and enhancements to public health resources and infrastructure [4].

### Future directions and considerations

Several important ethical and procedural questions related to informed consent in psychiatric research require further consideration and empirical study [93]. For example: How should fluctuations in decisional capacity be handled? How can participant advocates be incorporated into the consent process? What degree of risk is reasonable in studies that likely include large numbers of individuals who won’t go on to develop psychosis? It should be noted, however, that concerns and questions regarding decisional capacity and competence are not unique to psychiatric research and unrelated factors such as medical illness and low education levels can impact the informed consent process independently of psychiatric status. As in the general population, there is great heterogeneity in decisional capacity and risk reasoning among individuals at risk for mental disorders and stigmatizing attitudes about the appropriateness of psychiatric research [94] should not be allowed to unduly hinder important scientific initiatives. Duration of untreated psychosis is one of the most highly modifiable illness characteristics and the fact that community practitioners already are prescribing antipsychotic medication to at-risk individuals [32] highlights the tremendous need for this type of work to proceed quickly and with utmost concern for the rights and welfare of the study participants so that the care for these individuals can be informed by the highest quality research.

As this research endeavor becomes increasingly internationalized, researchers must carefully attend to issues related to cultural differences in understanding mental illness, research and consent and international differences in regulatory requirements. Especially in low-income countries and communities, care must be taken to balance the benefits accrued to the research team and those accrued to participants and to avoid exploitation of research participants that can result from taking advantage (either intentionally or unintentionally) of power differentials among countries and among individuals [4].

## Conclusions

Schizophrenia represents a personal tragedy for those afflicted, a source of sorrow for family members, and a costly public health concern. Available interventions cannot cure this devastating disorder, but early intervention may help shift the illness trajectory from chronic disability to recovery of function. Consequently, scientific efforts to discern the earliest developmental stages of schizophrenia in the prodromal period must move forward. Studies conducted to date represent a laudable step toward the identification of illness onset markers and suggest treatment approaches that may prevent or delay the transition to active psychosis. Future investigations are expected to chart presymptomatic pathways with greater precision, hopefully uncovering pathogenic mechanisms that will be targeted in the coming generation of prevention studies. Including diverse cultural perspectives will result in a more comprehensive knowledge base and will require investigators to work across international boundaries. These studies will continue to rely on the participation of competent research subjects who must understand, appreciate, and act on the elements of informed consent presented in this report.
